# Urokinase prophylactic anticoagulation in children with nephrotic syndrome: a multicenter retrospective cohort study

**DOI:** 10.1186/s12882-024-03502-4

**Published:** 2024-02-26

**Authors:** Zhen Wang, Nan Wang, Ruyue Chen, Hanyun Tang, Qiang Lin, Xiaozhong Li

**Affiliations:** 1 Department of Renal Immunology, Children’s Hospital of Soochow University, Soochow, JiangSu China; 2 Department of Pediatrics, Zibo Maternal and Child Health Hospital, Zibo ShanDong, China

**Keywords:** Urokinase, Prophylactic, Primary nephrotic syndrome, Children, Thromboembolism

## Abstract

**Objective:**

To analyze the clinical effect of urokinase on the prevention of thrombosis in children with primary nephrotic syndrome.

**Methods:**

A total of 370 children diagnosed with primary nephrotic syndrome (PNS) in the Children’s Hospital of Soochow University and Zibo Maternal and Child Health Hospital from January 2018 to December 2022 were selected as the research objects. The patients were divided into a urokinase adjuvant therapy group and non-urokinase adjuvant therapy group according to the application of drugs. The clinical data of the children were collected, including sex, age, drug application, bleeding during treatment, and telephone follow-up, to record whether thromboembolism occurred in the acute stage and remission stage. The clinical pattern of PNS, renal biopsy, histopathological type, and related laboratory indexes before and after treatment were recorded.

**Results:**

A total of 313 patients were treated with urokinase and 57 patients were not. More thrombotic events was observed in non-urokinase group compared to the urokinase group(2 versus 0 episodes, *p* = 0.02). The thrombotic events observed included one patient had pulmonary embolism combined with right ventricular thrombosis, and another had intracranial venous thrombosis. More minor bleeding events occurred in urokinase group compared to the non-urokinase group(7 versus 1 episodes, *p* = 1.0). No major bleeding events occurred in either group.

**Conclusion:**

The rational prophylactic use of urokinase anticoagulation in children with PNS can prevent the formation of thromboembolism and has good safety.

## Background

Nephrotic syndrome (NS) is one of the most common renal diseases in children, accounting for 25% of renal diseases. The main clinical manifestations are massive proteinuria accompanied by hypoproteinemia, edema, and hyperlipidemia [1].

The pathogenesis of NS is still unclear. Changes in “podocyte molecules” are considered to be the essence of the development of proteinuria, and changes in the properties of the glomerular capillary filtration barrier caused by the immune response are the primary factors of proteinuria. In the past, most scholars believed that the occurrence of nephrotic syndrome was related to T cells. However, the efficacy of rituximab and other specific B-cell suppressive agents has challenged the T-cell origin hypothesis [2]. Recently, the discovery of circulating anti-nephrin antibodies has provided further evidence for the autoimmune etiology of minimal change disease (MCD) [3]. Thromboembolism is a serious complication of NS, and previous studies have found that the incidence of primary nephrotic syndrome (PNS) thrombosis in children is about 2–5% [4, 5], which is similar to the results of our previous study [6]. The symptoms of thromboembolism have different manifestations according to the site of occurrence. High-risk pulmonary artery thrombosis often presents with the triad of chest pain, hemoptysis, and dyspnea [7–10]. The most common symptom of intracranial thrombosis is headache. Deep vein thrombosis is a common cause of limb pain, swelling, and difficulty in walking [11]. Severe costoabdominal pain, costoovertebral angle tenderness, hematuria, proteinuria, and renal dysfunction are the main manifestations of renal vein thrombosis. If bilateral renal vein thrombosis occurs rapidly, it might develop into oliguric acute renal failure [12, 13]. At present, there is still a lack of standard thrombolytic therapy after thrombosis, and once thromboembolism is formed, the prognosis is often poor; therefore, reasonable preventive anticoagulant therapy is particularly important. In 1884, Virchow first proposed three key factors for thrombosis: A hypercoagulable state, vascular endothelial injury, and hemodynamic factors [14]. Large amounts of small molecular weight proteins, including coagulation factors IX, XI, and XII, are spilled into the urine because of glomerular permeability changes. At the same time, the concentrations of anticoagulant factors also decrease, including antithrombin III (ATIII), protein C, protein S, and tissue factor pathway inhibitor. In contrast, the concentrations of high molecular weight proteins increase, including coagulation factor I, II, V, VII, VIII, X, Von Willebrand factor (vWF), and fibrinogen. This is because these macromolecule proteins are not easily lost and, as a compensatory mechanism, there is increased hepatic protein synthesis. Under a hypercoagulable state, clotting function is activated and, at the same time, the fibrinolytic system is activated [5]. In addition, the role of endothelial injury in thrombosis has received increased attention. Endothelial cells mainly play an anticoagulant role under physiological conditions [15]; however, when the body is in a state of nephropathy, the increase in oxygen free radicals in the body, hyperlipidemia, and the application of glucocorticoids and immunosuppressants can cause endothelial cell damage [16]. In a injury state, endothelial cells synthesize and release tissue factor, thromboxane A2 (TXA2), plasminogen activator inhibitor 1 (PAI-1), and vWF, which participate in the activation of internal and external coagulation pathways [17].

A number of observational studies have found that the high risk factors of thrombosis in children with NS mainly include the age of onset (≥ 12 years) [18, 19], indwelling intravenous catheter [18, 20–22], complicated by infection [23], persistent severe hypoproteinemia (< 20 g/l) [22, 24], hyperlipidemia [20, 21, 25, 26], hyperfibrinogenemia [19, 22], platelets (Plt) > 300 × 10^9^/l [19, 27], glucocorticoids application [19, 28], ATIII < 80% [20, 22, 24], Ddimer > 1 mg/l [24, 29], and diuretics application [19, 28]. The occurrence of thrombosis often indicates a poor prognosis, and many cases of disability and even death have been reported in the literature at home and abroad [30–35]. However, the potential adverse reactions to anticoagulants make the prophylactic use of anticoagulants in children with NS controversial.

At present, most clinicians are still cautious about the prophylactic use of anticoagulant drugs in children with PNS, and the 2021 Kidney Disease Improving Global Outcomes (KDIGO) guidelines only proposes the use of anticoagulant drugs in adults with membranous nephropathy. The present study retrospectively analyzed the clinical results of urokinase (UK) application as an anticoagulant drug in the treatment of PNS in children in the Children’s Hospital of Soochow University and Zibo Maternal and Child Health Hospital from 2018 to 2022, aiming to provide guidance for the prevention of thromboembolism in children with PNS.

## Methods

### Design and inclusion

The subjects of this study were patients diagnosed with PNS admitted to the Department of NephroImmunology, Children’s Hospital of Soochow University and the Department of Pediatrics, Zibo Maternal and Child Health Hospital, from January 2018 to December 2022. This clinical study was a retrospective study. The information provided by the patients was anonymized before analysis, and every effort was made to protect the privacy of the data. Informed consent was waived. The study was approved by the Medical Ethics Committee of Children’s Hospital of Soochow University. The diagnostic criteria for PNS included: (1) Proteinuria (≥ 50 mg/kg/d) or a protein/creatinine ratio (mg/mg) in the morning urine ≥ 2.0; (2) hypoproteinemia (< 25 g/L), (3) hyperlipidemia (total cholesterol > 5.7 mmol/L); and (4) significant edema. The inclusion criteria were: patients less than 18 years old. In multiple hospitalizations, only the occurrence of embolism events were recorded, and in multiple hospitalizations without embolism, the data of the first hospitalization was recorded. The exclusion criteria were: hereditary, secondary nephrotic syndrome; hospital stay less than 1 week; patients with incomplete data and patients lost to follow-up. The patients were followed up for at least 5 months.

### Intervention

A total of 370 subjects were enrolled. The 313 patients in the urokinase treatment group were treated with urokinase at a dose of 1500–2000U/kg for 1–2 weeks in addition to basic treatment of nephrotic syndrome, excluding contraindication of urokinase use. There were 57 patients in the non-urokinase treatment group. Some patients were treated with low molecular weight heparin (LMWH) at a dose of 100 IU/(kg.d) for 2 to 4 weeks.

### Outcome

The baseline characteristics and clinical results of the study subjects were obtained by reviewing their medical records, and the sex, age, previous history of thrombosis, drug application, and bleeding during hospitalization were recorded. The patients were followed up by telephone to record whether they had thromboembolism in the acute stage and remission stage. Patients with clinically suspected thromboembolism were confirmed by imaging examination. The clinical pattern of NS, renal biopsy, and histopathological type were recorded. The laboratory indicators (including serum albumin, cholesterol, D-dimer, triglyceride, urea nitrogen, serum creatinine, fibrinogen, and urine protein) were collected during the acute phase of hospitalization and at the first time of remission. Bleeding events were classified as major bleeding events or minor bleeding events depending on whether blood transfusion or blood products was required or not.

### Data analysis

SPSS26.0 statistical software (IBM Corp., Armonk, NY, USA) was used for the statistical analysis. Normal distributed parameters were expressed as the mean ± standard deviation (SD), and nonnormal distributed parameters were expressed as the median [interquartile range (IQR)]. Chisquared, Fisher’s exact, and T tests were used to perform comparisons between groups.

## Results

**Fig. 1:**
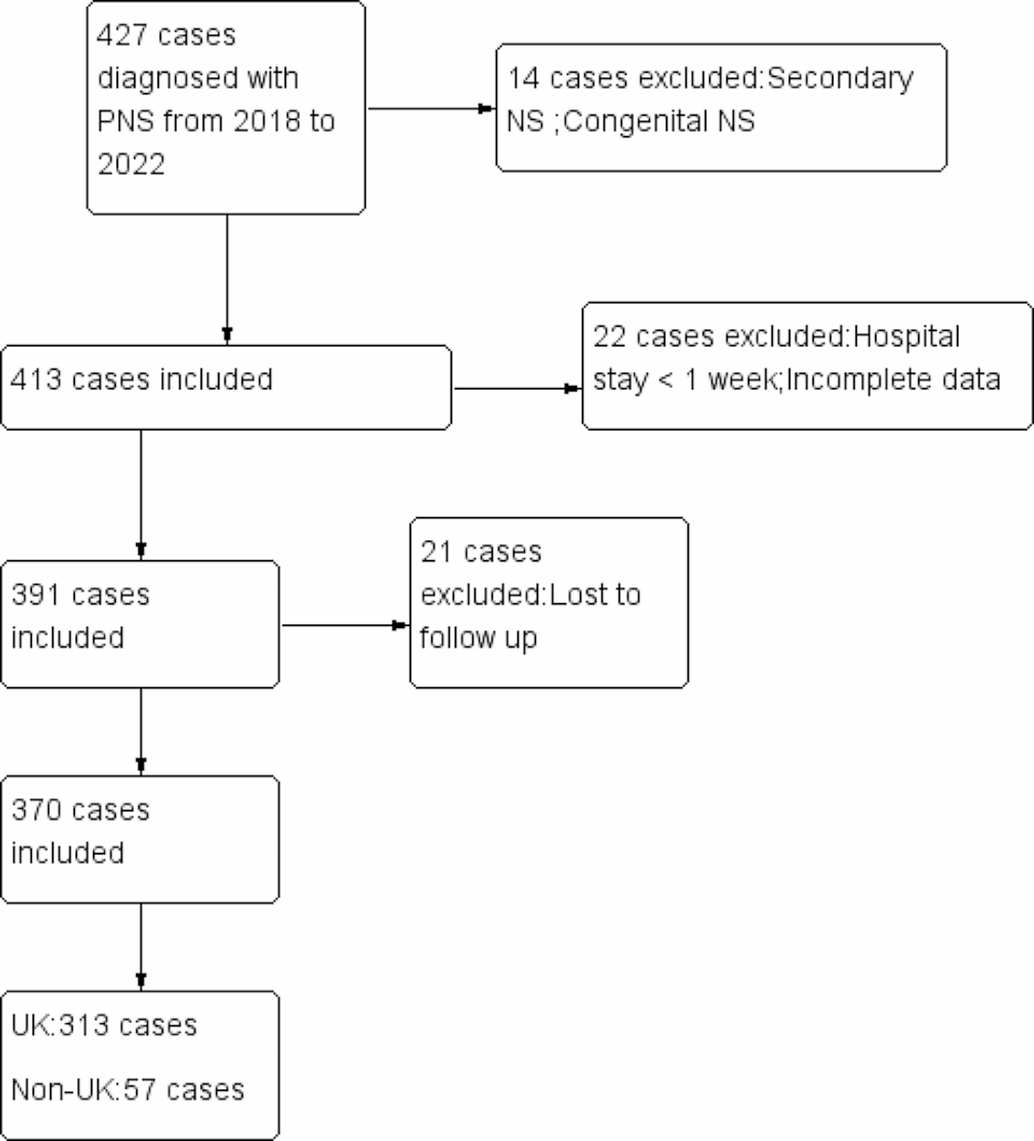
Flowchart of the inclusion and exclusion of the patients. PNS, primary nephrotic syndrome; UK, urokinase

A total of 370 children were included in the study (Fig. 1). Among them, 313 patients were treated with urokinase (UK) and antiplatelet therapy, 57 patients with antiplatelet therapy alone or combined with LMWH.

The distribution of clinical pattern of NS: In the UK group, 266 cases were diagnosed with steroid sensitive-infrequently relapsing NS, 18 cases were diagnosed with steroid sensitivefrequently relapsing NS, 13 cases were diagnosed with steroid-resistant NS, and 16 cases were diagnosed with steroid sensitive-steroid dependent NS. In the non-UK group, 40 cases were diagnosed with steroid sensitive-infrequently relapsing NS, 7 cases were diagnosed with steroid sensitive-frequently relapsing NS, 3 cases were diagnosed with steroid-resistant NS, and 7 cases were diagnosed with steroid sensitive-steroid dependent NS. Based on the biopsy results, in the UK group, of the 41 patients who underwent renal biopsy, mesangial hypercellularity was found in 33, MCD in 3, membranous nephropathy (MN) in 2, focal segmental glomerulosclerosis (FSGS) in 2, and membranous proliferative glomerulonephritis (MPGN) in 1. In non-UK group, of the six patients who underwent renal biopsy, mesangial hypercellularity was found in four, MCD in one, and FSGS in one. All patients received antiplatelet therapy. In the nonUK group, three patients received anticoagulant therapy with LMWH. No significant differences were found between the two groups in terms of sex, age, and renal biopsy results (Table 1).

**Table 1:** Baseline characteristics of the included patients

	UK (*N* = 313)	Non-UK (*N* = 57)	*P*-value
Sex, Male (n, %)	211(66.7%)	35(61.4%)	0.38
Age (year)	5.7 ± 3.8	6.8 ± 4.4	0.06
Clinical pattern of NS (n, %)			0.01
Steroid sensitive-infrequently relapsing	266(85.0)	40(70.2)	
Steroid sensitive-frequently relapsing	18(5.75)	7(12.3)	
Steroid sensitive-steroid dependent	16(5.11)	7(12.3)	
Steroid-resistant	13(4.15)	3(5.26)	
Biopsy diagnosis (n, %)			0.61
MCD	3(1)	1(1.8)	
mesangial hypercellularity	33(10.5)	4(9.8)	
MN	2(4.9)	0(0)	
FSGS	2(4.9)	1(1.8)	
MPGN	1(2.4)	0(0)	
no Biopsy	272(86.9)	51(89.5)	
LMWH	0 (0)	3(1)	0.03
Median serum albumin(g/L) Hypoproteinemia(n, %)	20.1 ± 5.1 272(86.9)	21.8 ± 5.9 44(77.2)	0.02 0.07
Fibrinogen(ug/L)	5.7 ± 1.4	6.1 ± 1.8	0.06
D-Dimer(ug/L)	1282.9 ± 1338.8	910.8 ± 1174.1	0.03

The comparison between UK and Non-UK was made using T-test, Fischer’s test, or a Chi-squared test

There were no significant differences in cholesterol, blood urea nitrogen, serum creatinine, triglyceride, and fibrinogen between the two groups at admission (*p* > 0.05). Patients in the UK group had lower serum albumin levels (*p* = 0.02) and higher D-dimer levels (*p* = 0.03) on admission. There was no significant difference in serum albumin, creatinine, and urea nitrogen between the two groups during the remission phase. Cholesterol and triglyceride levels in the UK group were higher than those in non-UK treatment group (*p* < 0.05) (Table 2).

**Table 2:** Clinical features in the different phases of PNS

	Clinical phase	UK	Non-UK	*P*-value
Median serum albumin (g/L)	acute phase	20.1 ± 5.1	21.8 ± 5.9	0.02
	remission or partial remission phase	29.2 ± 5.4	30.3 ± 4.3	0.31
Median serum cholesterol (mmol/L)	acute phase	10.7 ± 3.0	10.9 ± 3.0	0.63
	remission or partial remission phase	7.2 ± 2.3	6.6 ± 1.4	0.03
Median serum triglyceride (mmol/L)	acute phase	2.9 ± 2.1	2.8 ± 1.8	0.90
	remission or partial remission phase	2.2 ± 1.3	1.8 ± 1.1	0.02
Median serum urea nitrogen(mmol/L)	acute phase	4.7 ± 3.0	5.1 ± 1.9	0.34
	remission or partial remission phase	4.0 ± 1.3	4.1 ± 1.2	0.57
Median serum creatinine (µmol/L)	acute phase	35.6 ± 17.3	34.4 ± 18.6	0.64
	remission or partial remission phase	31.9 ± 11.7	34.6 ± 10.4	0.11

The comparison between the UK and Non-UK groups was made using a T-test

There was no thrombotic event in the UK group, although seven minor bleeding events occurred, all of which were small bleeding points on the skin. Thrombotic events occurred in two patients (*p* = 0.02) who did not receive UK therapy, and both patients received antiplatelet therapy only. In one case, pulmonary embolism and right ventricular thrombus occurred 4 months after the diagnosis of NS. The other case was intracranial venous thrombosis, which occurred 3 years after the diagnosis of NS and the day after the recurrence of NS. The early detection of thrombosis meant that both patients recovered well after active anticoagulant and thrombolytic therapy, and no sequelae occurred. There was one minor bleeding event (*p* = 1.0), comprising a small bleeding spot on the skin. The patient with bleeding was treated with LMWH and antiplatelet drugs (Table 3).

**Table 3:** Thromboembolic events (TEs) and bleeding episodes

	UK (*n* = 313)	Non-UK (*n* = 57)	*p*-value
Thromboembolic events (TE), n (%)	0 (0)	2 (3.5)	0.02
Bleeding episodes, n (%)	7 (2.2)	1 (1.8)	1.0
Major, n (%)	0 (0)	0 (0)	
Minor, n (%)	7 (2.2)	1 (1.8)	1.0

## Discussion

Our previous study found that the incidence of thrombosis in children with PNS was 4.9% [6]. In this study, no thrombosis was found in the UK treatment group, which was superior to the nonUK treatment group. There were only seven minor bleeding events, which was not statistically significant compared with the one that occurred in the non-UK treatment group. The results indicated that urokinase as an adjuvant therapy in children with PNS is safe and effective in preventing thromboembolism.

The lack of randomized trials means that there is little evidence in the literature regarding the optimal prophylactic agent for PNS or its dosage. Available evidence consists mainly of case studies or retrospective cohort studies; however, they represent local treatment protocols rather than universally accepted treatment strategies. Low molecular weight heparin and warfarin are the most commonly used prophylactic anticoagulants in patients with NS. A recent study by Kelddal et al. [36] found that anticoagulant therapy with LMWH and warfarin can reduce the risk of thromboembolic events (TE) in patients with NS; however, they increase the risk of bleeding, even major bleeding, and the risk is higher when combined with antiplatelet therapy. In a retrospective study of 143 patients treated with anticoagulants conducted by Medjeral-Thomas et al. [37], one patient with MCD developed pulmonary embolism 6 days after initiation of a prophylaxis regimen, and one patient with MN developed pulmonary embolism 5 days after presentation. Neither of the two patients were treated with prophylactic anticoagulants for more than 1 week. In that study, major bleeding occurred in two patients treated with aspirin and one with LMWH. Direct Acting Oral Anticoagulants (DOACs) have been recognized in clinical practice because of their rapid effect, stable and predictable anticoagulant effect, fewer food and drug interactions, no routine anticoagulation monitoring, and low bleeding risk. Their efficacy has also been demonstrated in nonvalvular atrial fibrillation and Deep Vein Thrombosis (DVT). However, it is unclear whether DOACs can match or even exceed conventional anticoagulants in patients with NS. Recently, Van Meerhaeghe et al. [38] found that patients with NS treated with apixaban anticoagulation had a reduced risk of TE without an increased risk of bleeding. However, there are few reports on the use of apixaban in children with NS.

Urokinase have several advantages over heparin and warfarin. In particular, UK has a rapid onset and offset of action, with predictable dosing, which precludes the need for routine coagulation monitoring in the general population. However, it should not be used in patients with acute hemorrhage, old cerebral infarction, or intracranial tumors. As a traditional thrombolytic drug, UK can dissolve fibrin by activating plasminogen, converting it into plasmin, thus dissolving thrombosis and preventing thrombosis and anticoagulation. However, the protective effect of UK on patients with NS goes beyond that. After binding to its cell surface receptor, urokinase plasminogen activator surface receptor (uPAR), urokinase can communicate intracellular signals through vitronectin and integrin binding, and mediate cell anti-apoptosis through the phosphoinositide 3-kinase (PI3K)/protein kinase B (PKB) and mitogen activated protein kinase (MAPK)/ extracellular signal-regulated kinase (ERK) pathways [39–42]. It can protect vascular endothelial cells from apoptosis and inhibits activation of the coagulation pathway. It has also been reported that urokinase can inhibit cell apoptosis through inhibitor of nuclear factor kappa-B kinase subunit alpha (IKKα)-nuclear factor kappa-B (NF-κB)-inhibitors of apoptosis (IAPs) pathway after binding to uPAR [43]. In addition, urokinase binds to uPAR and activates focal adhesion kinase (FAK), which in turn activates the Ras-Raf-MEK-ERK1/2 signaling pathway. Activated ERK1/2 further activates NF-κB and activator protein 1 (AP-1), eventually leading to enhanced mRNA transcription and protein expression of matrix metalloproteinase 9 (MMP9). Increased expression of MMP-9 can degrade the extracellular matrix and slow down the occurrence of renal interstitial fibrosis [44].

In our study, There were no significant differences in cholesterol, urea nitrogen, serum creatinine, triglyceride, and fibrinogen between the two groups on admission (*p* > 0.05). Patients in the UK group had lower serum albumin levels and higher D-dimer levels at admission. Persistent hypoproteinemia and elevated D-dimer levels are high risk factors for NS complicated with thromboembolism [22, 24, 29]; therefore, the group receiving urokinase might be more prone to TE, suggesting that the benefit of UK in preventing TE might be greater than observed. Table 2 shows that UK had no obvious advantage in terms of recovery from NS. After treatment, hyperlipidemia persisted in both groups, indicating that high risk factors for thromboembolism still existed after the patients entered the remission period. Thromboembolism mostly occurred within 6 months after the onset of NS, and some studies reported that the median time of thromboembolism was about 70.5 days after diagnosis [45]. In the present study, pulmonary embolism with right ventricular thrombus occurred 4 months after the diagnosis of NS in one patient. This suggested that the hypercoagulable state persists for a long time and does not disappear as the patient enters the remission period, and especially when high-risk factors such as infection exist, the incidence of thromboembolism will increase [23]. Barbour et al. [46] found that the median time to venous thromboembolic events (VTE) in patients with glomerulonephritis was 272 days, with only 70% of VTE episodes occurring within the first 2 years. The other case developed intracranial venous thrombosis with seizures as the initial symptom, which occurred 3 years after the diagnosis of NS and was complicated by thrombosis when the patient’s NS recurred. It was suggested that the recurrence of NS also has the possibility of thrombosis. Park et al. [47] described a case of thrombosis with steroid dependent MCD. The patient presented with diffuse abdominal pain and vomiting, and an enhanced abdominal computed tomography (CT) scan revealed diffuse portal vein, spleen, and superior mesenteric vein thrombosis.

In recent years, studies have proposed the concept of subclinical venous embolism or asymptomatic thrombosis [19, 48], and then questioned the incidence of thromboembolism in NS reported in previous studies. Therefore, it is possible that this type of thrombotic event was missed in our study. In 2017, the International Society on Thrombosis and Haemostasis (ISTH) established a research group to redefine this type of thrombosis as clinically unsuspected venous thromboembolic events, and published their results in 2020, which showed that this type of thrombus does not have a significant adverse effect on the prognosis of patients [49].

Our study is limited by its retrospective design, the small study sample size, and the low incidence of thrombosis. In addition, the study subjects were from two different medical centers, which means that there might be differences in clinical practice that could affect the final results. However, the baseline data of the patients in the two groups were similar, and the same inclusion and exclusion criteria were applied; therefore, the results are considered to be representative.

## Conclusion

Based on real-world data, the results of the present study suggest that UK is effective and safe to prevent thromboembolism in children with NS. With the increasing acceptance of immunosuppressive agents by clinicians and the promotion of biological agents in clinical practice, pediatric NS has basically achieved multi-target precise treatment; however, complications, such as thrombosis, have not been solved. Until recently, there were still reports of thromboembolism in children with NS. By searching the China National Knowledge Infrastructure (CNKI), Wanfang database, and PUBMED database, we found that three cases of NS complicated by TE were reported in 2022 [50–52],five cases in 2021 [53–57], and six cases in 2020 [58–62]. Unreported thromboembolic events might be even more striking. Lurking behind the numbers is the disability and even mortality associated with concomitant thromboembolism. Therefore, we should prevent complications, especially thromboembolism, while paying attention to kidney injury in patients with nephropathy.

## Data Availability

The datasets generated and/or analysed during the current study are available from corresponding author on reasonable request.

## Abbreviations

NS: Nephrotic syndrome
UK: Urokinase
LMWH: Low molecular weight heparin
SD: Standard deviation
IQR: Interquartile range
MCD: Minimal change disease
MN: Membranous nephropathy
FSGS: Focal segmental glomerulosclerosis
MPGN: Membranous proliferative glomerulonephritis
TE: Thromboembolism
PNS: Primary nephrotic syndrome
DVT: Deep venous thrombosis
DOACs: Direct Acting Oral Anticoagulants
VTE: Venous thromboembolism
CT: Computed tomography
ISTH: International Society on Thrombosis and Haemostasis
CNKI: China National Knowledge Infrastructure
uPAR: Urokinase plasminogen activator surface receptor
PI3K: Phosphoinositide 3-kinase
PKB: Protein kinase B
MAPK: Mitogen activated protein kinase
ERK: Extracellular signal-regulated kinase
IKKα: Nuclear factor kappa-B kinase subunit alpha
NF-κB: Nuclear factor kappa-B
IAPs: Inhibitors of apoptosis
FAK: Focal adhesion kinase
MMP9: Matrix metalloproteinase 9
AP-1: Activator protein 1
